# Rewiring of adult neural networks - learning from structural plasticity across multiple scales

**Published:** 2026-10-13

**Authors:** Michael Fauth, Han Lu, Jannik Luboeinski, Johanna Senk, Simone Albani, Valentina Armuzza, Paolo Carloni, Richmond L. Crisostomo, Hermann Cuntz, Ulrich Egert, Eugenio F. Fornasiero, Tobias Gemmeke, Claus C. Hilgetag, Sabine M. Hölter, Patrick Inoue, Björn Kampa, James C. Knight, Susanne Kunkel, Thomas Nowotny, Samora Okujeni, Nicola Palomero-Gallagher, Mariia Popova, Petra Ritter, Giulia Rossetti, Michael Schirner, Marc Spehr, Leon Stefanovski, Gaia Tavosanis, Agata Witkowska, Felix Wolf, Willem Wybo, Sandra Diaz-Pier, Maximilian Lenz, Abigail Morrison, Florian Röhrbein, Stefan Rotter, Johannes Schemmel, Christian Tetzlaff, Andreas Vlachos

## Abstract

Synaptic connections are continuously formed and eliminated even in adult neuronal networks. This process - called structural plasticity - allows networks to change their topology to learn and adapt to their environment or compensate for lesions. Yet, in contrast to synaptic plasticity, structural plasticity is less well studied and understood. We here present the current state of knowledge as well as open problems in the field along three guiding questions: (1) What regulates structural plasticity in biological neural networks? (2) What are the functional consequences and advantages of structural plasticity in adult neural networks? (3) How can our understanding of structural plasticity be translated into clinical and technological applications? We argue that understanding structural plasticity is a problem involving multiple temporal and spatial scales. Solving this problem thus requires collaborative efforts of experimentalists, theoreticians as well as computational modelers, and has the potential for comprehensive neuroscientific insights that can guide the development of medical applications, artificial neural networks as well as neuromorphic computing.

